# Therapeutic Ultrasound Modulates Cell Proliferation and Proinflammatory Cytokine Levels in Osteoarthritic Chondrocytes

**DOI:** 10.1111/jcmm.70257

**Published:** 2025-01-12

**Authors:** Ahmet Çagdas Yilmaz, Hasan Toktas, Sefa Celik, Serkan Sen

**Affiliations:** ^1^ Department of Physical Medicine and Rehabilitation Afyonkarahisar Health Sciences University Afyonkarahisar Turkey; ^2^ Department of Medical Biochemistry Afyonkarahisar Health Sciences University Afyonkarahisar Turkey; ^3^ Ataturk Vocational School of Health Services, Department of Medical Laboratory Techniques Afyonkarahisar Health Sciences University Afyonkarahisar Turkey

**Keywords:** chondrocytes, osteoarthritis, proinflammatory cytokines, therapeutic ultrasound

## Abstract

The development and progression of osteoarthritis (OA) are believed to involve inflammation. This study aimed to investigate the effects of applying therapeutic ultrasound (US) to human osteoarthritic chondrocytes in continuous and pulsed modes on cell proliferation and proinflammatory cytokine levels. Human osteoarthritic chondrocytes (HC‐OA 402OA‐05a) were proliferated in appropriate media and then seeded into culture plates. The plates were grouped and exposed to underwater continuous, pulsed and control US at 0.1 W/cm^2^ and 1 MHz for 10 min daily for 10 days. Cell viability/proliferation was assessed using the MTT assay, total protein was measured by ELISA and cytokine levels per protein were determined. Cells were photographed using microscopic analysis. Both continuous and pulsed US groups showed a significant increase in viability compared to the control group. No significant difference was found between the continuous and pulsed US groups for IL‐1β, TNF‐α and IL‐6 levels. Both groups showed significant cytokine reduction compared to the control group. For IL‐17 and IL‐32 levels, both US groups had reduced cytokine levels compared to the control group, but the results were not significant. Underwater US at 0.1 W/cm^2^ and 1 MHz stimulated cell proliferation and reduced proinflammatory cytokine levels in osteoarthritic chondrocyte cell cultures. This study extensively focused on proinflammatory IL levels, and the meaningful results may inspire future in vivo/in vitro studies. While adapting in vitro data to in vivo conditions poses challenges, our results could guide future in vivo studies and clinical applications.

## 1 Introduction

Osteoarthritis (OA) is the most common form of arthritis. OA is characterised by joint cartilage loss and synovial inflammation. It also leads to swelling, pain and loss of movement in the joints, typically starting at an older age and involving degenerative processes [1].

Inflammation, genetic predisposition, environmental factors, trauma and biochemical changes contribute to OA pathogenesis. Evidence shows that cartilage and bone metabolism change and differentiate in OA, and the cartilage matrix shows signs of ageing. Epidemiological and biological evidence reflects a connection between the development of OA and age‐related inflammation or ‘inflamm‐aging’. Sources of age‐related proinflammatory mediators that may contribute to OA include peripheral sources such as adipose tissue, which increases with age, and local production within joint tissues [2, 3, 4].

OA development and progression are believed to involve inflammation, even in early disease stages. Epidemiological studies have shown a connection between the presence of reactive or inflammatory synovium and the progression of tibiofemoral cartilage damage. Therefore, secreted inflammatory mediators such as proinflammatory cytokines can be critical mediators of the impaired metabolism of joint tissue involved in OA. Interleukin (IL)‐1β, tumour necrosis factor (TNF)‐α and IL‐6 appear to be the main proinflammatory cytokines involved in OA pathophysiology, as well as others, such as IL‐15, IL‐17, IL‐18 and IL‐21, leukaemia inhibitory factor and chemokines [5]. IL‐17 is a proinflammatory cytokine subclass that participates in tissue inflammation and degradation by inducing the expression of matrix metalloproteinases (MMPs) and other proinflammatory mediators such as IL‐1β, IL‐6 and TNF‐α in chondrocytes and synovial fibroblasts. This process can contribute to cartilage degradation and synovial infiltration in OA. In addition, the proportion of IL‐17‐secreting cells in the blood of patients with OA was reported to be higher than that of healthy controls, and high IL‐17 levels were found in the synovial fluid of patients with OA [6], IL‐32 is a proinflammatory cytokine that induces other cytokines contributing to inflammation, including IL‐1β, IL‐6 and TNF‐α. Gui et al. [7, 8] reported that IL‐32 was highly expressed in the synovial tissues of patients with rheumatoid arthritis, whereas it was weakly expressed in synovial tissues obtained from patients with OA.

In OA, nonpharmacological treatment methods include patient education and preventive measures, recognising factors that cause excessive joint loading, preventing obesity and applying physical medicine and rehabilitation. Pharmacological treatments include simple analgesics such as paracetamol and nonsteroidal anti‐inflammatory drugs (NSAIDs), opioids, intra‐articular injections and viscosupplementation. Surgical treatments are also performed.

In recent years, research on the therapeutic effects of ultrasound (US) as a physical therapy agent on cartilage and bone tissue has accelerated. Many in vitro, in vivo and clinical studies have shown that US positively affects cartilage healing. These indicate that low‐intensity US applications may induce a healing effect on the cartilage tissue. This study aimed to investigate the effects of therapeutic US on proinflammatory cytokines, which are believed to be released in the early stages of inflammation.

## 2 Methods

This study was conducted in the Laboratory of the Department of Medical Biochemistry, Faculty of Medicine, Afyonkarahisar Health Sciences University, and was approved by the Clinical Research Ethics Committee of Afyonkarahisar Health Sciences University, dated 4 June 2021.

### 2.1 Procurement and Characteristics of Chondrocytes

Human‐derived osteoarthritic cells (HC‐OA 402OA‐05a) were used. The cells were obtained from Sigma & Aldrich (MA, USA). The osteoarthritic chondrocyte cell line was derived from a 56‐year‐old Caucasian individual with knee OA. A healthy control group was not used in the experiments.

### 2.2 Culturing of Chondrocytes

The basal medium for the cell line was Dulbecco's modified eagle medium (DMEM). Thirteen millilitres of DMEM was added to a 75 cm^2^ cell culture flask and incubated at 37°C with 5% CO_2_ for 30 min. After seeding, the culture medium was left in the incubator for proliferation. The medium was changed every 48–72 h. All conditions were maintained in a sterile environment using a class II biosafety cabinet.

### 2.3 Seeding and Culturing of Chondrocytes in Culture Plates

Cells were detached using trypsin/EDTA, neutralised with DMEM and centrifuged at 400 rpm for 10 min. After discarding the supernatant, cells were resuspended in DMEM, counted and checked for viability (> 97%). The cells were then seeded into 75‐cm^2^ flasks and 96‐well plates for further experiments.

### 2.4 Application of Therapeutic US to Chondrocytes

**FIGURE 1:**
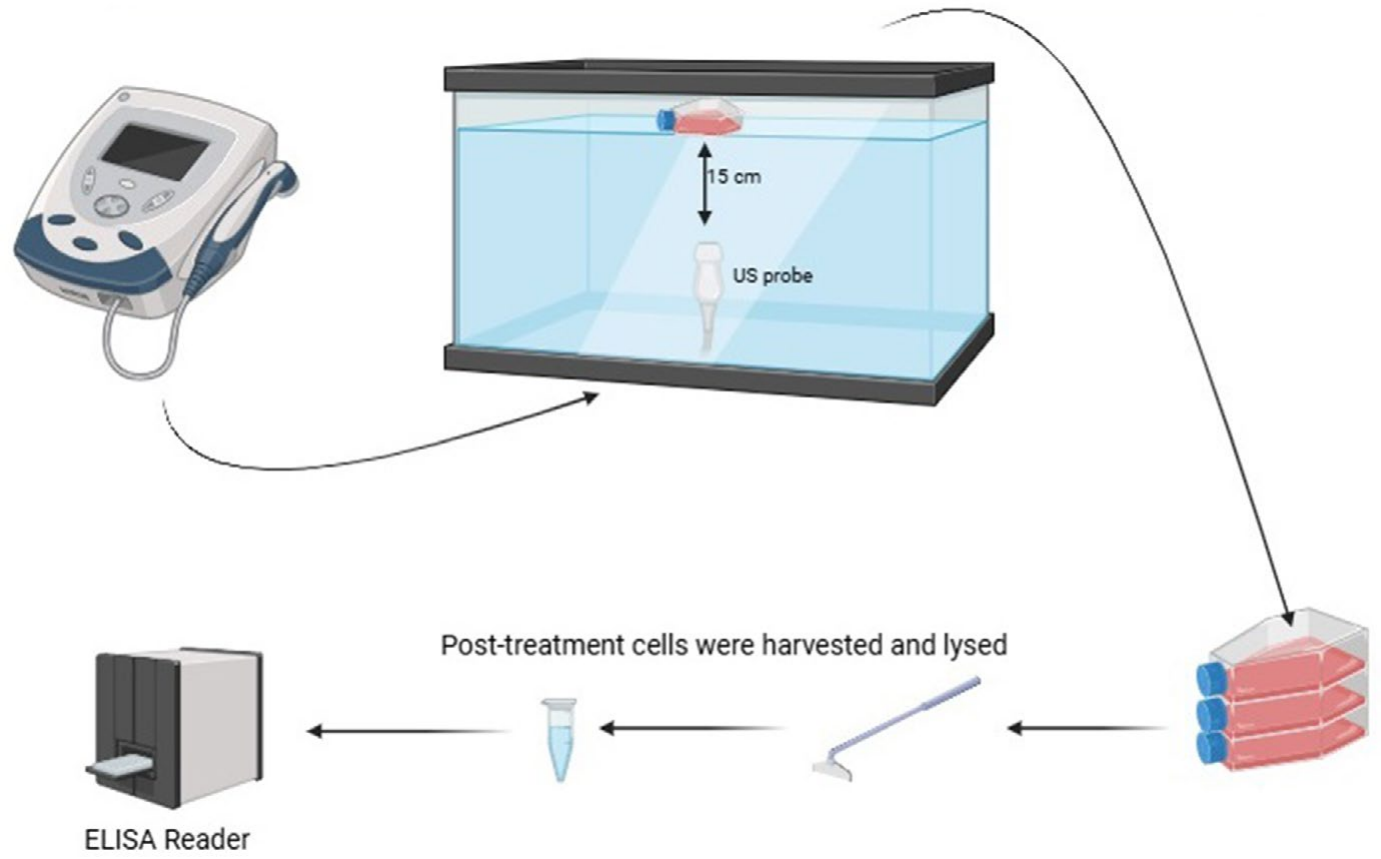
Setup of the experimental method.

This study was conducted in two phases. In the first phase, nine 75‐cm^2^ cell culture flasks were divided into three groups of three: control (sham mode), continuous (0.1 W/cm^2^–1 MHz, 100% duty cycle) and pulsed (0.1 W/cm^2^–1 MHz, 20% duty cycle) US groups. In the second phase, 96‐well cell culture plates were assigned to the control, continuous and pulsed US groups (Figure 1).

The Chattanooga Group US Service (2776, USA‐2012) was used in this study, as it is employed for therapeutic purposes in our daily practice. This US device allows applications at the lowest intensity of 0.1 W/cm^2^. The device was planned to operate in three different modes at 0.1 W/cm^2^ and 1 MHz intensity and frequency: control (sham mode), continuous and pulsed (20% duty cycle).

**FIGURE 2:**
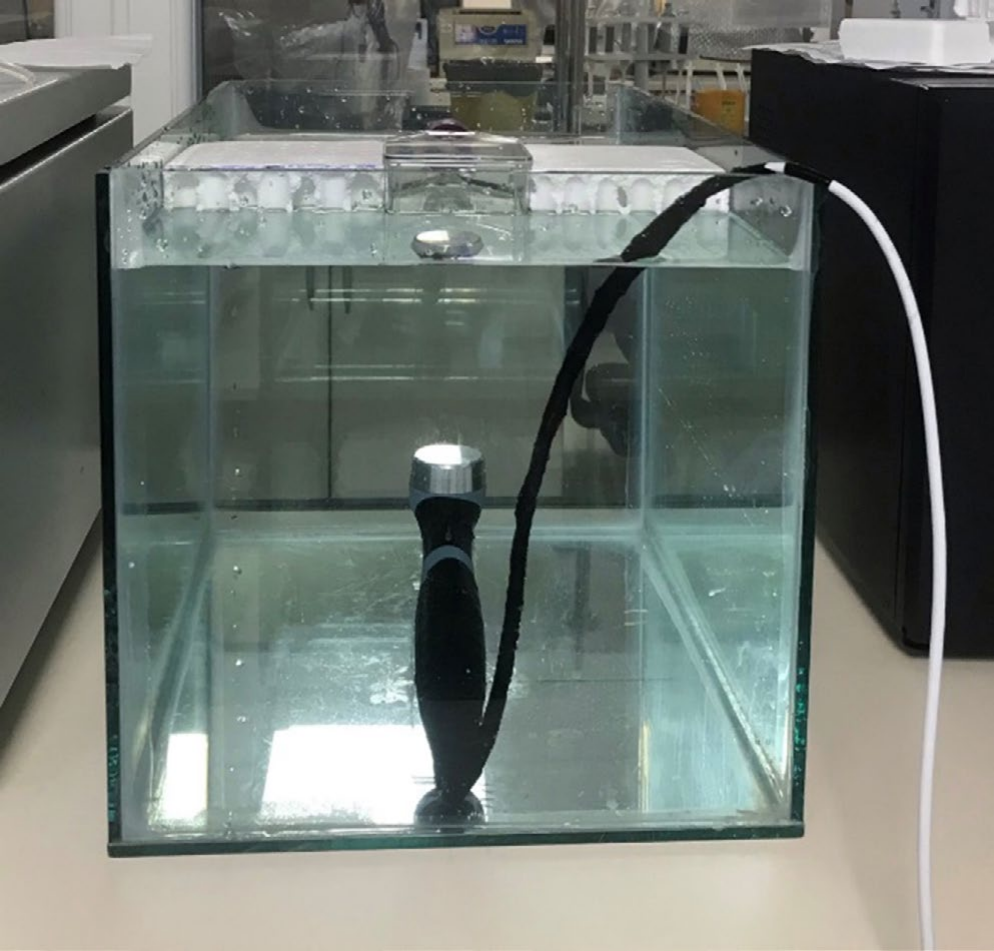
Administration of ultrasound therapy.

A custom‐made glass aquarium was used, considering the angle at which the US probe would be placed. The probe was positioned in the aquarium and fixed after sterilisation. Sterile distilled water at 37°C was added up to the marked level in the aquarium. The 75‐cm^2^ cell culture flasks were fixed in a predetermined area within the aquarium, ensuring a 15‐cm distance between the cell culture flasks and the probe, and the US device was activated (Figure 2) [9]. Initially, the continuous mode was applied, followed by the pulsed mode, with each session lasting 10 min. In the control group, cell culture flasks were fixed above the aquarium for 10 min with the US device in sham mode. After each phase, the water temperature was checked with a thermometer. If the temperature dropped, the water was replaced with hot water and allowed to rest, and sterile distilled water at 37°C was reestablished. This experimental setup was designed based on the setup used by Tien et al. [9].

### 2.5 Preparation of Cell Lysates and Total Protein Measurement

After incubations, the cells that adhered to the surface of the culture flasks were detached by adding trypsin and gently shaking the flasks. The suspensions were collected in capped tubes and centrifuged at 400 rpm for 5 min at 25°C to obtain pellets. The pellets were washed with PBS, recentrifuged and a cell lysis buffer was then added. The supernatant containing the dissolved proteins was obtained by centrifuging at 12,000 rpm for 10 min at 4°C to remove detergent‐insoluble proteins. Total protein levels were measured using the bicinchoninic acid (BCA) protein assay kit [10]. The readings were taken at 562 nm using an Epoch Biotek enzyme‐linked immunosorbent assay (ELISA) reader, following the standard protocol.

### 2.6 IL Level Measurements by ELISA

Levels of IL‐1β, IL‐6, IL‐17, IL‐32 and TNF‐α were measured using commercial ELISA kits. ELISA strips were prepared and run according to the manufacturer's instructions for each cytokine. Readings were taken at 450 nm using an Epoch Biotek ELISA reader.

### 2.7 3‐(4,5‐Dimethyl‐2‐Thiazolyl)‐2,5‐Diphenyl‐2H‐Tetrazolium‐Bromide (MTT) Cell Viability Assay

An MTT dye (5 mg/mL) was added to each well of the 96‐well cell culture plates. After 2 h of incubation, the MTT dye was removed, and 200 μL of dimethyl sulfoxide (DMSO) was added to each well and incubated for another 10 min. The colour change was measured at 570 nm using an ELISA plate reader. The viability of the control group was considered 100%, and that of the experimental cells was expressed as a percentage. The MTT assay is a valuable method for in vitro evaluation of cell proliferation and cytotoxicity, providing information based on the number of cells and mitochondrial activity per cell. Its advantages include speed, ease, safety and cost‐effectiveness [11, 12].

### 2.8 Microscopic Analysis

Using an inverted microscope, cell counting and calculation of live/dead ratios in the cell cultures were performed by an experienced biochemist. Microscopic images were captured with a digital camera.

### 2.9 Statistical Analysis

Statistical data analysis was performed using GraphPad PRISM version 8.0.1. To compare multiple variables within and between groups, the one‐way ANOVA and Tukey's multiple‐comparison test were used.

## 3 Results

### 3.1 Cell Viability and Proliferation With the MTT Assay

**FIGURE 3:**
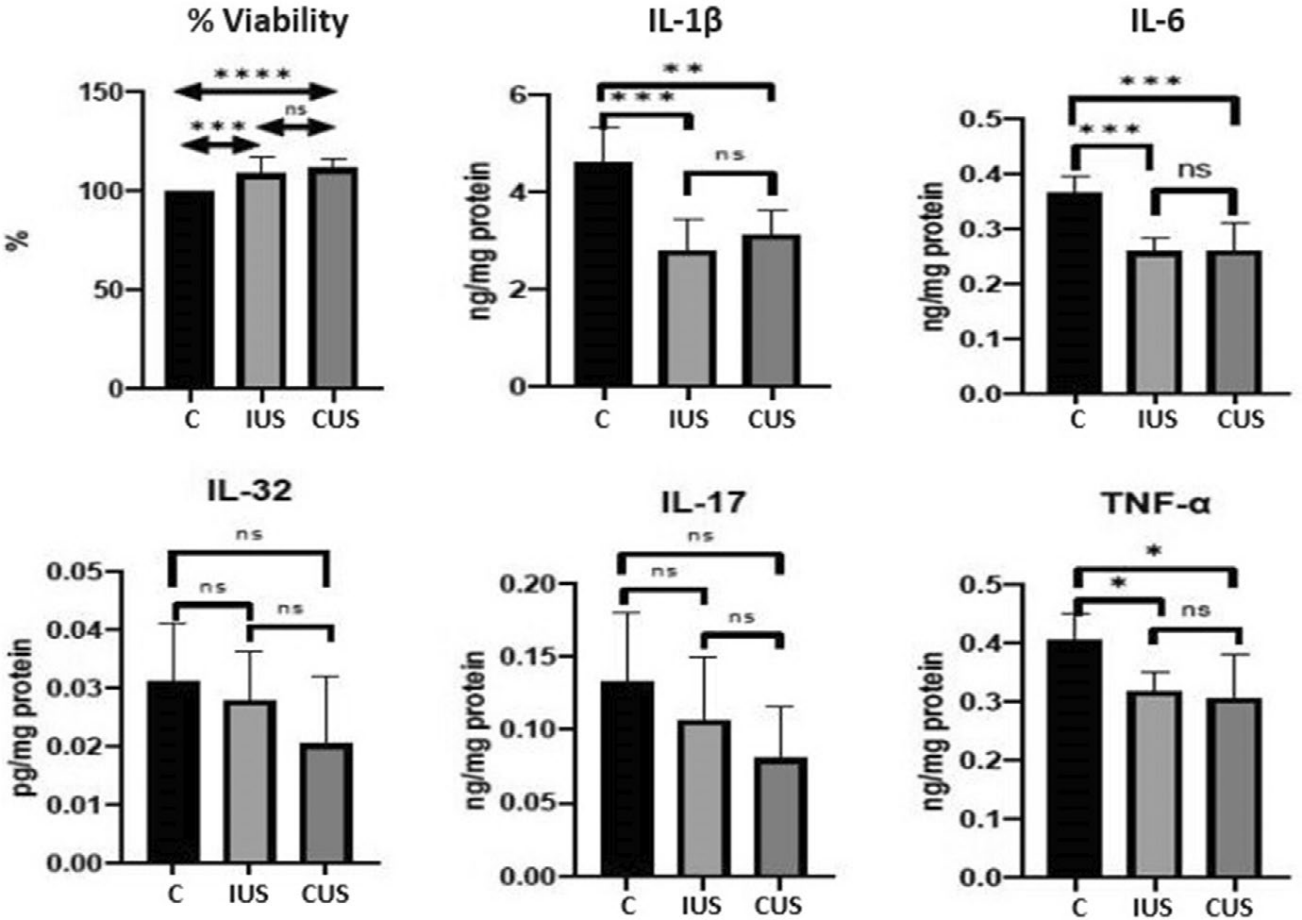
Statistical significance values for % viability and proinflammatory cytokine levels across the groups. C, control; CUS, continuous US; IUS, intermittent US. Asterisks (*) indicate statistically significant differences between groups (*p* < 0.05). The number of asterisks represents the degree of significance, with more asterisks indicating a lower *p*‐value and a stronger statistical difference.

No significant difference was found between the continuous and intermittent US groups. However, a significant difference was observed in favour of the treatment groups compared with the control group. The increase in viability in the continuous US group compared with that in the control group had a *p*‐value of ≤ 0.0001, and the increase in viability in the intermittent US group compared with that in the control group had a *p*‐value of ≤ 0.001. As the MTT assay also reflects cellular proliferation, these results can be interpreted accordingly. US application did not harm the cells, on the contrary, it increased cell viability. Relevant data are shown in Table 1 and Figure 3.

**TABLE 1:** Mean values of proinflammatory interleukins and % viability by treatment groups and significance according to one‐way ANOVA.

	IL‐1β (ng/mg protein)	IL‐6 (ng/mg protein)	TNF‐α (ng/mg protein)	IL‐17 (ng/mg protein)	IL‐32 (pg/mg protein)
Control US	4.603^a^	0.365^a^	0.404^a^	0.132^a^	0.030^a^
Intermittent US	2.786^b^	0.257^b^	0.317^b^	0.106^b^	0.027^b^
Continuous US	3.127^b^	0.259^b^	0.304^b^	0.079^c^	0.020^c^
*p*‐value	0.0004	0.0002	0.0102	Ns	ns
	**Control US**	**Intermittent US**	**Continuous US**		
% Viability	100^a^	108.72^b^	111.44^b^		
*p*‐value	< 0.0001				

*Note: p*‐value: Statistical significance according to the one‐way ANOVA. Different superscripts between groups indicate that there is a statistical difference between these groups.

Abbreviations: ANOVA, analysis of variance; continuous US group, cells treated with continuous ultrasound; intermittent US group, cells treated with intermittent ultrasound.

### 3.2 Determination of IL Levels by ELISA

In IL‐1β, a significant decrease was observed in favour of the intermittent US group compared with the control group (*p* ≤ 0.001). A significant decrease was also observed in favour of the continuous US group compared with the control group (*p* ≤ 0.01). However, no significant difference was seen between the two treatment groups.

For IL‐6, a significant difference was observed between the continuous US and control groups, in favour of the continuous group (*p* ≤ 0.001). The same significant difference was observed in the comparison between the intermittent US and control groups (*p* ≤ 0.001). However, no significant difference was found between the continuous and intermittent US groups.

The TNF‐α results were similar to those of IL‐1β and IL‐6. A significant decrease was observed in favour of the continuous US group compared with the control group (*p* ≤ 0.05). A significant decrease was also observed in favour of the intermittent US group compared with the control group (*p* ≤ 0.05). However, no significant difference was found between the intermittent and continuous US groups.

Regarding IL‐17, although a decrease was observed in both continuous and intermittent US groups compared with the control group, this decrease was not significant. Similarly, no significant difference was found between the continuous and intermittent groups, although IL‐17 levels decreased in the continuous group.

For IL‐32, results were similar to those of IL‐17. No significant decrease was observed in both continuous and intermittent US groups compared with the control group. Although the decrease in the continuous US group was greater than that in the intermittent group, the difference was not significant.

### 3.3 Microscopic Analysis

Immediately before preparing cell lysates at the end of treatment, samples from the control, continuous and intermittent US groups were placed in a Neubauer chamber and stained with trypan blue. As a result, an average of four cells were observed per field in the control group, whereas an average of eight cells were observed per field in both the intermittent and continuous US groups. These data quantitatively indicate an increase in cell count in the treatment groups compared with the control group.

Table 2 qualitatively demonstrates how the organisation of cells changed throughout the treatment. As shown in the table, noticeable changes in cell organisation were found in the treatment groups, with a marked increase in cell density and arrangement, reflecting the detailed effects of the treatment methods.

**TABLE 2:** Photographs of ultrasound groups during treatment at ×4 magnification.

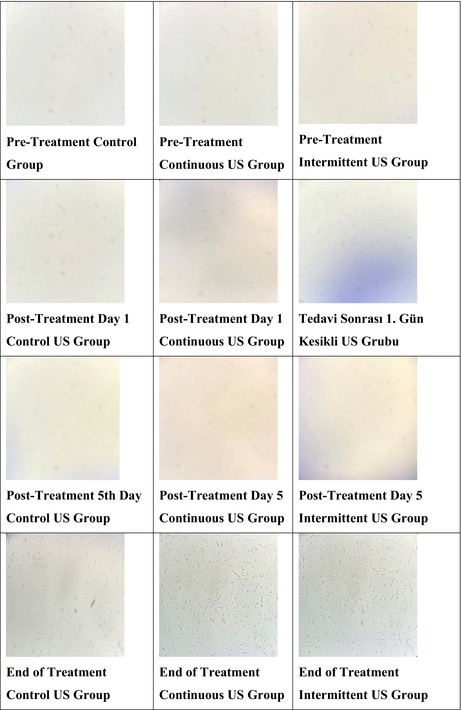

## 4 Discussion

OA is the most common joint disease. Its pathogenesis involves genetic predisposition, environmental factors, trauma, cartilage damage and changes in the subchondral bone, and synovial inflammation was also implicated. The treatment options include analgesics such as acetaminophen, NSAIDs, anti‐inflammatory drugs, viscosupplementation, serotonin–norepinephrine reuptake inhibitors and opioids. For advanced OA where medical treatments fail to improve symptoms and daily living activities are impaired, surgical options are considered.

Despite the relatively broad spectrum of pharmacological and nonpharmacological treatment modalities available for OA, no treatment method demonstrated high evidence levels aimed at disease control. Therefore, research has shifted towards this direction. Numerous studies on therapeutic US for OA treatment have been conducted, with several still ongoing. However, evidence on whether the nonthermal effects of US have disease‐modifying properties is still insufficient. Low‐intensity, intermittent US might stimulate cell proliferation and reduce proinflammatory cytokine levels. However, no standard has been established in the studies conducted so far. Therefore, this study was designed to investigate the effects of therapeutic US on chondrocyte proliferation and proinflammatory cytokines.

In the literature review, studies on low‐intensity therapeutic US have explored cell viability, proliferation, gene expression and pro‐ and anti‐inflammatory cytokines. However, many of these studies exhibit variations in US intensity, frequency, application methods and distances. For instance, Oliviera et al. compared low‐intensity intermittent US (LIUS) at various intensities and duty cycles in a fibroblast culture model. Cells were subjected to US for 2 min at 24, 48 and 72 h, and cell viability was assessed after each treatment. The group subjected to 0.5 W/cm^2^ intensity and 10% duty cycle showed viability superior to those of the other groups at 48 h [13]. Another study divided the cells in a fibroblast culture into five groups and treated them with various LIUS intensities at 24, 48, 72 and 96 h. After treatment, cell proliferation was significant in the 0.6 W/cm^2^ group with a 20% duty cycle [14]. Mostafa et al. [15] used human gingival fibroblasts and treated them with 1.5 MHz, 30 mW/cm^2^ US for 5 or 10 min over 28 days. No significant difference in cell viability was observed between the groups.

In another study using continuous LIUS, human osteoarthritic chondrocytes were cultured on alginate beads and treated with 0, 100, 200 and 300 mW/cm^2^ intensities, continuously for 15 days at 10 min per day. Cells were observed on days 2, 7 and 15. Tripan blue staining showed a significant increase in cell viability after 15 days, with the highest increase observed at 200 mW/cm^2^. However, no significant difference in cell proliferation was noted at 7 days [16]. In a study using neonatal rat chondrocytes, cells were treated with 0, 50 and 120 mW/cm^2^, 1 MHz continuous US for 10 min daily for 5 days. Proliferation and proteoglycan synthesis were measured on days 1, 3 and 5. Although proliferation increased over time, no significant differences were observed between the groups, and this finding also applied to proteoglycan synthesis [17]. Suzuki et al. [18] used rat clonal cells, passaged them and plated them in 96‐well cell culture plates. They applied 30 mW/cm^2^, 1.5 MHz LIUS for 14 days at 20 min daily. Cell viability was assessed using a different colorimetric method from the MTT test, and no difference in viability was found between the treated and control groups.

Sang et al. [19] used mouse chondrocytes and treated them with 50 or 100 mW/cm^2^ LIUS for 10 min. The number of chondrocytes increased compared with that in the control group, and IL‐6, IL‐8 and TNF‐α levels in the synovial fluid were significantly reduced. Another study on fibroblast‐like synoviocytes demonstrated that IL‐1β can induce NO, MMP‐1, MMP‐3 and PGE2 and that this induction can be abolished by LIUS through NF‐κB [20]. In another continuous LIUS study with fibroblasts, groups were categorised as control, 0.2 W/cm^2^ with 10% duty cycle and 0.2 W/cm^2^ with 20% duty cycle at 1 and 3 MHz frequencies. Treatment was applied for 24–48 h. Cell viability and *IL‐6* and *VEGF* expression levels were assessed. Increased cell proliferation and decreased *IL‐6* expression levels were observed in the 0.5 W/cm^2^, 3 MHz group, whereas *VEGF* expression levels increased [21]. Another study involving human osteoblasts, fibroblasts and monocytes used various doses of continuous and LIUS (5, 15, 30 and 50 mW/cm^2^) applied from a 5‐cm distance in water. In that study, continuous US and LIUS stimulated cell proliferation slightly increased IL‐1β levels in all cell types but did not show differences in IL‐6 and TNF‐α levels. Particularly, IL‐8 and fibroblast growth factor production increased in osteoblasts, and VEGF levels increased in all cell types [22]. In a fibroblast study, MTT was used to assess viability, and flow cytometry was employed to evaluate various pro‐ and anti‐inflammatory cytokines in the control group, 0.2 W/cm^2^ group with 10% duty cycle and 0.2 W/cm^2^ group with 20% duty cycle. Each group received 2 min of US treatment at 24, 48 and 72 h. Viability was assessed with MTT, and significant increases in viability and IL‐6 and VEGF levels were observed, particularly in the 0.2 W/cm^2^ group with 10% duty cycle at 48 h [23]. A recent study confirmed that LIUS regulates YAP phosphorylation and reduces YAP and P‐RIPK1 binding, significantly preventing IL‐1β‐induced chondrocyte inflammation and autophagy damage [24].

In vivo studies on therapeutic US were also conducted locally and globally. In the placebo‐controlled, double‐blind, randomised controlled trials of Taşçıoğlu et al. 90 knees of patients with OA were examined, and patients were divided into three groups: group 1 received continuous US at 2 W/cm^2^, group 2 received the same intensity with 20% duty cycle intermittent US and group 3 received closed‐mode US. Treatments were applied for 5 min daily over 10 working days, with significant improvements in visual analogue scale scores in both intermittent and continuous US groups compared with the control group. In addition, improvement was greater in the intermittent US group than in the continuous group. However, no significant reduction in Western Ontario and McMaster University Osteoarthritis Index scores was found in the continuous group compared with the control group [25]. Rodríguez‐Grande et al. [26] evaluated the effects of a 10‐day application of therapeutic US at 2.2 W/cm^2^ with a 20% duty cycle on patients with knee OA and concluded that US significantly reduced pain levels from day 5 onwards, and this reduction continued until the end of treatment, indicating that US can be recommended for knee OA treatment. Owing to the in vitro design, difficulties are common in translating the data to in vivo conditions. However, the results obtained are likely to guide in vivo studies and clinicians.

## 5 Conclusion

Both in vivo and in vitro studies show variations in US intensities, frequencies, media, application distances and methods used. These differences have led to varying results despite similar goals. Consequently, no consensus has been established on the use of US therapy for OA. The lack of consensus and the need for a better understanding of cellular processes in OA highlight the importance of this study, which evaluates the effects of low‐intensity therapeutic US on proinflammatory IL levels. This study is significant given its comprehensive examination of proinflammatory ILs and is expected to inspire future in vivo and in vitro studies.

### 5.1 Limitations

This study investigated the effects of therapeutic ultrasound (US) on osteoarthritic chondrocyte cell cultures. However, the use of an in vitro model limits the direct applicability of the findings to in vivo conditions. The study focused only on specific proinflammatory cytokine levels, potentially overlooking other significant inflammatory markers. Additionally, the effects of different variations of US parameters and longer treatment durations were not explored. Therefore, further studies are required to generalise these findings to a broader population. Furthermore, while detailed seeding and culturing protocols were included to ensure reproducibility, the complexity of the method presentation may have introduced unnecessary intricacies, which should be streamlined in future studies for clarity.

## Author Contributions

**Ahmet Çagdas Yilmaz:** conceptualization (equal), data curation (equal), funding acquisition (equal), methodology (equal), writing – original draft (equal). **Hasan Toktas:** conceptualization (equal), funding acquisition (lead), investigation (equal), methodology (equal), project administration (lead), resources (equal), supervision (lead), writing – original draft (equal). **Sefa Celik:** data curation (equal), funding acquisition (equal), methodology (equal). **Serkan Sen:** investigation (equal), methodology (equal), writing – review and editing (lead).

## Ethics Statement

This research was carried out in accordance with the Declaration of Helsinki and received approval from the Noninvasive Clinical Research Ethics Committee of Afyonkarahisar Health Sciences University on June 4, 2021 (approval number: 2021/7).

## Conflicts of Interest

The authors declare no conflicts of interest.

## Data Availability

The datasets utilised and/or analysed in this study will be made available by the corresponding author upon reasonable request.

## References

[1] G. Musumeci , F. C. Aiello , M. A. Szychlinska , M. di Rosa , P. Castrogiovanni , and A. Mobasheri , “Osteoarthritis in the XXIst Century: Risk Factors and Behaviours That Influence Disease Onset and Progression,” International Journal of Molecular Sciences 16 (2015): 6093–6112. 25785564 10.3390/ijms16036093 PMC4394521

[2] M. Rahmati , G. Nalesso , A. Mobasheri , and M. Mozafari , “Aging and Osteoarthritis: Central Role of the Extracellular Matrix,” Ageing Research Reviews 40 (2017): 20–30. 28774716 10.1016/j.arr.2017.07.004

[3] V. Francisco , T. Pérez , J. Pino , et al., “Biomechanics, Obesity, and Osteoarthritis. The Role of Adipokines: When the Levee Breaks,” Journal of Orthopaedic Research 36, no. 2 (2018): 594–604. 29080354 10.1002/jor.23788

[4] S. Wakale , X. Wu , Y. Sonar , et al., “How Are Aging and Osteoarthritis Related?,” Aging and Disease 14, no. 3 (2023): 592–604. 37191424 10.14336/AD.2022.0831 PMC10187698

[5] A. I. S. Jrad , M. Trad , W. Bzeih , G. El Hasbani , and I. Uthman , “Role of Pro‐Inflammatory Interleukins in Osteoarthritis: A Narrative Review,” Connective Tissue Research 64, no. 3 (2023): 238–247. 36541851 10.1080/03008207.2022.2157270

[6] Y. H. Lee and G. G. Song , “Association Between IL‐17 Gene Polymorphisms and Circulating IL‐17 Levels in Osteoarthritis: A Meta‐Analysis,” Zeitschrift für Rheumatologie 79 (2020): 482–490. 31664512 10.1007/s00393-019-00720-2

[7] R. de Albuquerque , E. Komsi , I. Starskaia , U. Ullah , and R. Lahesmaa , “The Role of Interleukin‐32 in Autoimmunity,” Scandinavian Journal of Immunology 93 (2021): e13012. 33336406 10.1111/sji.13012

[8] M. Gui , H. Zhang , K. Zhong , Y. Li , J. Sun , and L. Wang , “Clinical Significance of Interleukin‐32 Expression in Patients With Rheumatoid Arthritis,” Asian Pacific Journal of Allergy and Immunology 31 (2013): 73–78. 23517397

[9] Y. C. Tien , S. D. Lin , C. H. Chen , C. C. Lu , S. J. Su , and T. T. Chih , “Effects of Pulsed Low‐Intensity Ultrasound on Human Child Chondrocytes,” Ultrasound in Medicine & Biology 34 (2008): 1174–1181. 18359144 10.1016/j.ultrasmedbio.2007.12.019

[10] M. M. Bradford , “A Rapid and Sensitive Method for the Quantitation of Microgram Quantities of Protein Utilizing the Principle of Protein‐Dye Binding,” Analytical Biochemistry 72 (1976): 248–254. 942051 10.1016/0003-2697(76)90527-3

[11] F. Denizot and R. Lang , “Rapid Colorimetric Assay for Cell Growth and Survival. Modifications to the Tetrazolium Dye Procedure Giving Improved Sensitivity and Reliability,” Journal of Immunological Methods 89 (1986): 271–277. 3486233 10.1016/0022-1759(86)90368-6

[12] H. Wan , R. Williams , P. Doherty , and D. F. Williams , “A Study of the Reproducibility of the MTT Test,” Journal of Materials Science. Materials in Medicine 5 (1994): 154–159.

[13] P. D. D. Oliveira , D. A. Oliveira , C. C. Martinago , R. C. P. Frederico , C. P. Soares , and R. F. D. Oliveira , “Effect of Low‐Intensity Pulsed Ultrasound Therapy on a Fibroblasts Cell Culture,” Fisioterapia e Pesquisa 22 (2015): 112–118.

[14] R. F. Franco de Oliveira , D. A. A. Pires Oliveira , and C. P. Soares , “Effect of Low‐Intensity Pulsed Ultrasound on l929 Fibroblasts,” Archives of Medical Science 7 (2011): 224–229. 22291760 10.5114/aoms.2011.22071 PMC3258710

[15] N. Z. Mostafa , H. Uludaǧ , D. N. Dederich , M. R. Doschak , and T. H. El‐Bialy , “Anabolic Effects of Low‐Intensity Pulsed Ultrasound on Human Gingival Fibroblasts,” Archives of Oral Biology 54 (2009): 743–748. 19493525 10.1016/j.archoralbio.2009.04.012

[16] B. H. Choi , J. I. Woo , B. H. Min , and S. R. Park , “Low‐Intensity Ultrasound Stimulates the Viability and Matrix Gene Expression of Human Articular Chondrocytes in Alginate Bead Culture,” Journal of Biomedical Materials Research. Part A 79 (2006): 858–864. 16886219 10.1002/jbm.a.30816

[17] J. Parvizi , C. C. Wu , D. G. Lewallen , J. F. Greenleaf , and M. E. Bolander , “Low‐Intensity Ultrasound Stimulates Proteoglycan Synthesis in Rat Chondrocytes by Increasing Aggrecan Gene Expression,” Journal of Orthopaedic Research 17 (1999): 488–494. 10459753 10.1002/jor.1100170405

[18] A. Suzuki , T. Takayama , N. Suzuki , M. Sato , T. Fukuda , and K. Ito , “Daily Low‐Intensity Pulsed Ultrasound‐Mediated Osteogenic Differentiation in Rat Osteoblasts,” Acta Biochimica et Biophysica Sinica 41 (2009): 108–115. 19204827 10.1093/abbs/gmn012

[19] F. Sang , J. Xu , Z. Chen , Q. Liu , and W. Jiang , “Low‐Intensity Pulsed Ultrasound Alleviates Osteoarthritis Condition Through Focal Adhesion Kinase–Mediated Chondrocyte Proliferation and Differentiation,” Cartilage 13, no. 2 Suppl (2021): 196S–203S. 32281401 10.1177/1947603520912322 PMC8804760

[20] N. Zhang , B. Xu , R. Xing , et al., “Low‐Intensity Pulsed Ultrasound Inhibits IL‐1β‐Induced Inflammation of Fibroblast‐Like Synoviosytes via NF‐κB Pathway,” Applied Acoustics 167 (2020): 107384.

[21] L. D. Bertin , R. C. Poli‐Frederico , D. A. A. Pires Oliveira , et al., “Analysis of Cell Viability and Gene Expression After Continuous Ultrasound Therapy in L929 Fibroblast Cells,” American Journal of Physical Medicine & Rehabilitation 98 (2019): 369–372. 30489278 10.1097/PHM.0000000000001103

[22] N. Doan , P. Reher , S. Meghji , and M. Harris , “In Vitro Effects of Therapeutic Ultrasound on Cell Proliferation, Protein Synthesis, and Cytokine Production by Human Fibroblasts, Osteoblasts, and Monocytes,” Journal of Oral and Maxillofacial Surgery 57 (1999): 409–420. 10199493 10.1016/s0278-2391(99)90281-1

[23] P. D. de Oliveira Perrucini , R. C. Poli‐Frederico , D. A. de Almeida Pires‐Oliveira , et al., “Anti‐Inflammatory and Healing Effects of Pulsed Ultrasound Therapy on Fibroblasts,” American Journal of Physical Medicine & Rehabilitation 99 (2020): 19–25. 31335343 10.1097/PHM.0000000000001265

[24] C. Pan , F. Lu , X. Hao , et al., “Low‐Intensity Pulsed Ultrasound Delays the Progression of Osteoarthritis by Regulating the YAP‐RIPK1‐NF‐κB Axis and Influencing Autophagy,” Journal of Translational Medicine 22 (2024): 286. 38493143 10.1186/s12967-024-05086-x PMC10943805

[25] F. Tascioglu , S. Kuzgun , O. Armagan , and G. Ogutler , “Short‐Term Effectiveness of Ultrasound Therapy in Knee Osteoarthritis,” Journal of International Medical Research 38 (2010): 1233–1242. 20925995 10.1177/147323001003800404

[26] E. I. Rodríguez‐Grande , J. L. Osma‐Rueda , Y. Serrano‐Villar , and C. Ramírez , “Effects of Pulsed Therapeutic Ultrasound on the Treatment of People With Knee Osteoarthritis,” Journal of Physical Therapy Science 29 (2017): 1637–1643. 28932004 10.1589/jpts.29.1637 PMC5599837

